# Editorial: Global youth mental health crisis: understanding challenges and advancing solutions in psychopathology

**DOI:** 10.3389/fpsyg.2026.1819079

**Published:** 2026-04-01

**Authors:** Atika Khalaf, Roger C. Gibson

**Affiliations:** 1 The PRO-CARE Group, Faculty of Health Science, Kristianstad University, Kristianstad, Sweden; 2 Hind Bint Maktoum College of Nursing and Midwifery, Mohammed Bin Rashid University of Medicine and Health Sciences, Dubai Health, Dubai, United Arab Emirates; 3 Department of Community Health and Psychiatry, The University of the West Indies, Kingston, Jamaica

**Keywords:** cognitive mechanism, developmental transitions, internal emotional state, relational environment, societal and historical contexts, youth mental health crisis

The global youth mental health crisis represents one of the most pressing public health challenges of the early 21st century. This is reflected in the substantial increase in the global burden of mental disorders among adolescents over the last 30 years (Tian et al., 2025). This trend reflects the cumulative impact of intersecting societal, developmental, and contextual factors. Disease, e.g. the COVID-19 pandemic, economic uncertainty, armed conflict, rapid digitalization, and persistent social inequities are all examples of these factors.

This Research Topic was conceived to broaden awareness about the psychopathological issues that today's youth experience, including their mechanisms and determinants. It also aimed to identify possible directions for prevention, intervention, and policy that are sensitive to developmental and cultural contexts. The 11 contributions to the topic collectively provide a multidimensional perspective on youth mental health. The included studies span developmental stages, cultural settings, and methodological approaches, offering an integrated picture of how psychological distress emerges, is maintained, and can potentially be mitigated.

A unifying theme across several studies is the central role of relational environments in shaping mental health outcomes. Research focusing on children with chronic health conditions highlights how attachment patterns and relational security are closely associated with psychopathological symptoms, emphasizing the enduring influence of caregiving relationships on emotional development and regulation (Turin Drouet et al.). These early relational dynamics are not confined to childhood but extend into adolescence, where family processes interact with evolving social contexts. Evidence linking parental psychological control to cyberbullying victimization and nonsuicidal self-injury among boarding school adolescents illustrates how familial dynamics and peer-related digital experiences converge to heighten vulnerability (Wei et al.). These findings underscore that youth psychopathology is embedded within relational systems that span both offline and online environments.

Closely related to these relational processes are internal emotional and cognitive mechanisms that influence how young people respond to stress. Several contributions focus on depression and related internalizing symptoms, highlighting emotion regulation as a critical pathway. The moderated mediation model examining expressive suppression among college students demonstrates how maladaptive regulation strategies contribute to subthreshold depressive symptoms (Chen et al.). This work aligns with findings showing that family and peer influences shape adolescent psychological inflexibility, a construct associated with rigid thinking patterns and reduced adaptive coping (Liu et al.). Together, these studies suggest that interpersonal environments shape internal regulatory processes, which in turn influence susceptibility to depression and distress. Importantly, they also point to emotion regulation and flexibility as modifiable targets for early intervention.

Beyond interpersonal and intrapersonal factors, the Research Topic situates youth mental health within broader societal and historical contexts. A study conducted in Polish secondary schools during the simultaneous stressors of the COVID-19 pandemic and the war in Ukraine provides compelling evidence of elevated anxiety and depressive symptoms among adolescents, while also identifying social support as a protective factor (Badura-Brzoza et al.). This work illustrates how large-scale crises amplify existing vulnerabilities and create new stressors, reinforcing the need to conceptualize youth mental health within ecological frameworks that account for structural instability and collective trauma.

The importance of developmental transitions is another prominent theme. Adolescence and emerging adulthood are characterized by rapid psychological, social, and identity-related changes, making these periods particularly sensitive to stress. Psychodynamically-informed research offers insight into how adolescents conceptualize stress and coping, revealing intrapsychic processes that underlie both adaptive and maladaptive responses (Akın et al.). Complementing this theoretical perspective, qualitative research on transition preparation among children and adolescents with mental disorders in China captures lived experiences of readiness, uncertainty, and support needs during critical life changes (Miao et al.). These contributions highlight transitions as moments of heightened risk but also as opportunities for timely, developmentally informed intervention.

The Research Topic also addresses specific manifestations of psychopathology that have gained increasing global attention. Eating disorders among adolescents and young adults in Jamaica are examined within their cultural and sociodemographic context, drawing attention to the diversity of pathways through which disordered eating develops (Harrison et al.). This study expands the geographic and cultural scope of youth mental health research and demonstrates the importance of moving beyond Eurocentric models when addressing global mental health challenges.

In parallel with theory-driven and qualitative approaches, the Research Topic reflects growing interest in methodological innovation. The application of machine-learning techniques to identify risk profiles for depressive symptoms among Chinese college students demonstrates the potential of data-driven tools for early detection and targeted prevention (Yu et al.). While such approaches offer scalability and efficiency, their inclusion alongside relational, developmental, and qualitative studies highlights the importance of integrating technological innovation with contextual understanding.

Finally, research on emerging adulthood in a Russian sample examines how markers of adult identity relate to health outcomes, reinforcing the value of nuanced developmental frameworks that extend beyond adolescence (Mikhaylova). This contribution emphasizes that youth mental health trajectories do not abruptly end with adolescence but continue to evolve as individuals negotiate autonomy, responsibility, and identity.

Across the Research Topic, several cross-cutting insights emerge. First, youth psychopathology is shaped by multilevel and interacting determinants, encompassing individual emotional processes, family and peer relationships, digital environments, and societal conditions. Second, protective factors, including social support, adaptive coping, psychological flexibility, and emotion regulation, consistently buffer against distress, suggesting that strengthening these processes may yield broad mental health benefits. Third, the contributions highlight the necessity of cultural and contextual sensitivity, demonstrating that while common mechanisms exist, their expression and implications vary across settings. Finally, the integration of diverse methodologies points toward a future of youth mental health research that values both precision and person-centered care.

Collectively, this Research Topic advances a more comprehensive understanding of the global youth mental health crisis. It also offers insight into diverse strategies for overcoming the challenge that the crisis poses, and attaining equitable, effective, and sustainable solutions for improving youth mental health worldwide.
